# European Society for Swallowing Disorders FEES Accreditation Program for Neurogenic and Geriatric Oropharyngeal Dysphagia

**DOI:** 10.1007/s00455-017-9828-9

**Published:** 2017-08-04

**Authors:** R. Dziewas, L. Baijens, A. Schindler, E. Verin, E. Michou, P. Clave, The European Society for Swallowing Disorders

**Affiliations:** 1 Department of Neurology, University Hospital Münster, Münster, Germany; 2 Department of Otorhinolaryngology, Head and Neck Surgery, Maastricht University Medical Center, Maastricht, The Netherlands; 3 GROW-School for Oncology and Developmental Biology, Maastricht University Medical Center, Maastricht, The Netherlands; 4 Phoniatric Unit, Sacco Hospital Milano, Department of Biomedical and Clinical Sciences, University of Milano, Milan, Italy; 5 Physical and Rehabilitation Medicine, Physiology Department, Rouen University Hospital, Rouen, France; 6 GI Sciences, Division of Diabetes, Endocrinology and Gastroenterology, School of Medical Sciences, Faculty of Biology, Medicine and Health, University of Manchester, Manchester, Great Britain; 7 Centro de Investigación Biomédica en Red de Enfermedades, Hepáticas y Digestivas (CIBERehd), Hospital de Mataró, Universitat Autònoma de Barcelona, Mataró, Spain

## Introduction

The oropharyngeal swallow involves a rapid, highly coordinated set of neuromuscular actions beginning with lip closure and terminating with opening of the upper esophageal sphincter. The central coordination of this complex sensorimotor task uses a widespread network of cortical, subcortical, and brainstem structures. Many diseases and disorders affecting the central swallowing network or downstream peripheral nerves, muscles, and structures may result in an impaired oropharyngeal swallow, i.e., neurogenic oropharyngeal dysphagia (OD). In addition, aging is also associated with multifactorial changes of swallowing physiology for which the term presbyphagia has been coined [1]. OD has been reported in about 10–27% of older community dwelling residents [2–4]. In the nursing home, setting numbers are significantly higher and cross the 50% margin, which is similar to figures reported for older individuals admitted to hospital with a diagnosis of pneumonia [5]. Disease-specific prevalence data for OD are also substantial. Thus, disordered swallowing is reported in more than half of acute stroke patients and patients with traumatic brain injury, at least one-third of patients with Parkinson’s disease and dementia and a significant number of patients with neuromuscular disorders, such as amyotrophic lateral sclerosis and myasthenia gravis [6–11]. In view of the demographic shift, especially with increasing numbers of very old people, i.e., those aged over 85 years, these already alarming figures will further increase in the coming years since many underlying pathologies, particularly stroke, dementia and Parkinson’s disease, are age related [12]. It has been estimated up to 16 million US, 40 million EU and 8 million Japanese citizens require care for dysphagia. The clinical consequences of dysphagia are directly linked to the patient’s overall prognosis, and may include aspiration pneumonia, malnutrition, and dehydration. In the presence of disordered swallowing, mortality is increased and elevated rates of infectious complications have been reported for several medical conditions, such as stroke or Parkinson’s disease, but are also present in other patient populations [13]. In addition, older patients discharged from general hospitals with both dysphagia and malnutrition presented a mortality rate of 65.8% at 1 year follow-up [14].

## The ESSD FEES Accreditation Program

The above-mentioned data indicate that swallowing impairment is a nearly ubiquitous problem in the today’s medical world. Affected patients are either treated as outpatients or on an inpatient basis, where dysphagia is observed at all levels of care, from the general ward to the intermediate care/stroke unit and the intensive care unit.

To manage all the needs of patients with dysphagia, two complementary strategies should be established: first the development of well-coordinated multidisciplinary teams and dysphagia units in hospitals and second, the creation of a new professional profile, the deglutologist, to bring together knowledge and skills from different disciplines, to fully cover the diagnostic and therapeutic needs of our patients with dysphagia.

Usually, the first step in systematic evaluation of OD is a clinical swallowing evaluation. Patients that show any sign of dysphagia are referred for instrumental assessment if their condition allows it and if there is potential for change in the clinical management of the patient.

Together with the videofluoroscopic swallowing study (VFSS), fiberoptic endoscopic evaluation of swallowing (FEES) is today the most commonly chosen method for the instrumental assessment of swallowing [15]. In terms of day-to-day practicality, the merits of FEES are that (i) it can be performed at the bedside, thus facilitating examination of severely motor-impaired, bedridden or uncooperative patients, for example in the intensive care unit or the stroke unit; (ii) follow-up examinations can be performed at short notice and, if necessary, frequently; and (iii) oropharyngeal secretion management and efficacy of cleaning mechanisms, such as coughing and throat clearing, can be assessed simply and directly. During recent years FEES has been successfully applied in diverse patient populations and disease patterns. Among others, studies describing FEES in stroke and traumatic brain injury patients [16–18], patients with neurodegenerative (dementia, Parkinson’s disease) [9, 19, 20] and neuromuscular diseases (for example ALS, myasthenia gravis, myotonic dystrophy) [21–24] as well as head and neck cancers [25–27] have been published. FEES is also being increasingly applied in pediatrics [28–30], geriatrics, [31, 32] and intensive-care medicine [33, 34].

Despite the numerous possible applications of FEES and the undisputed need for qualified dysphagia assessment in this area of expertise, this technique is rarely taught systematically. For this reason, the European Society of Swallowing Disorders (ESSD) has decided to offer and organize an interdisciplinary pan-European training curriculum for FEES in neurogenic and geriatric dysphagia. This ESSD FEES accreditation program expands on the German FEES curriculum which is in use since 2014 [35, 36].

The ESSD is an international, nonprofit, multidisciplinary society, legally registered by the Department of Justice of the Generalitat de Catalunya, expedient nº 46,577, www.myessd.org. The legal framework for this initiative is defined in the aims of the society as recorded in the Articles of the Association, those directly relevant to this activity are the following:

- To promote care, education and research in swallowing physiology and swallowing disorders.
- To represent and promote the field to national and international authorities and societies and the European Union
- To create professional standards of practice and guidelines.
- To consolidate the Society as the society for dysphagia and swallowing disorders in Europe.

Also recorded in the Articles, the activities the ESSD will carry out to achieve these aims include the following:

- Promote the development of guidelines and best practice and recommended reading using the network of experts and associations
- Organize or promote training through workshops and courses
- Represent the field to the European Union and Commission and to international and national institutions and national governments

The ESSD provides an appropriate framework for developing this kind of professional training and accreditation, being both multidisciplinary and international, and has sought the endorsement of other medical societies. In addition to accreditation in FEES in neurogenic and geriatric dysphagia, the ESSD is developing professional accreditation programs in other methods of instrumental assessment such as VFSS and high resolution manometry. Given the need for instrumental assessment of dysphagia in neurogenic and geriatric patients, the multidisciplinary team management needed for dysphagia and the lack of training in FEES of the professions of the team, ESSD has the scope to be able to achieve this with the backing of other medical profession societies.

The ESSD FEES accreditation program pursues three aims: First, the definition of quality standards and systematic procedures, designed to guarantee the consistent performance of FEES throughout Europe. In the long run, the intended standardization of terminology, examination algorithms and interpretation of results will not only facilitate professional communication within a given hospital, but will also contribute to the optimization of understanding between the various sites involved in the treatment of an individual patient over time, e.g. acute clinic, rehabilitation clinic, outpatient care; Second, a formal accreditation program leading to a valorization of FEES as an independent, clinically relevant and sought-after qualification; Third, to provide a clear pathway to regulate the use of FEES by several professional domains, inside a multidisciplinary team, as practiced in other parts of the world and even in other disciplines.

The diagnostics and therapy of swallowing disorders are relevant to many disciplines. This training curriculum is therefore open to all clinicians with an interest in this topic. It also offers health care professionals the opportunity to acquire qualifications in the area of instrumental dysphagia assessment and to expand their range of activities.

At this point, attention should explicitly be drawn to the fact that the present curriculum addresses neurogenic and geriatric OD. Therefore, neither the diagnostics of structural changes in the mouth and throat (e.g. tumors or anatomical variants), nor the examination of swallowing disorders due to such ailments (e.g. structural changes after surgery or irradiation) are dealt with in detail in this training program and patients with any of these disorders should be referred to an otorhinolaryngologist or a phoniatrician.

Finally, this training curriculum will provide a thorough education for using FEES to evaluate the oropharyngeal swallow in neurological and geriatric patients and to establish a formal diagnosis of OD. It should be noted that management of OD requires a multidisciplinary team providing further knowledge and skills for complex cases and rehabilitation planning. The ESSD will evaluate whether more advanced training should be offered according to the feedback and experience from this basic level.

## Prerequisites

The following prerequisites have been defined for qualification in the area of FEES within this curriculum and will be reviewed by the ESSD:

- Two years of clinical practice focused on the care of neurological or geriatric patients is required for doctors and health care professionals. Three months of this period shall be completed in a neurological or geriatric department or a facility involving the care of these patients such as dysphagia or FEES units
- Along with the acquisition of the FEES certificate, the following requirements specific to each professional group must be fulfilled in order to attain the status of a FEES instructor: allied health professionals must be in possession of at least 5 years of experience in the area of diagnostics and therapy of neurogenic, geriatric OD. Physicians must have acquired a specialist title.

## Qualification Levels

The ESSD FEES accreditation program is divided into two stages: the **FEES certificate** and the **FEES instructor certificate**.

### FEES Certificate

The holder of a FEES certificate is accredited by the ESSD as able to perform FEES to assess neurogenic, geriatric OD, to prepare the related report and to define clinical consequences in collaboration with the multidisciplinary team. Key learning objectives of this educational step are (i) to safely pass the endoscope in a standard setting, (ii) to know and carry out the standard FEES protocol, (iii) to recognize the main findings of FEES (iv) to differentiate normal from pathological findings, (v) to recommend further treatment according to the findings and in the context of the management provided by the multidisciplinary team.

**Fig. 1:**
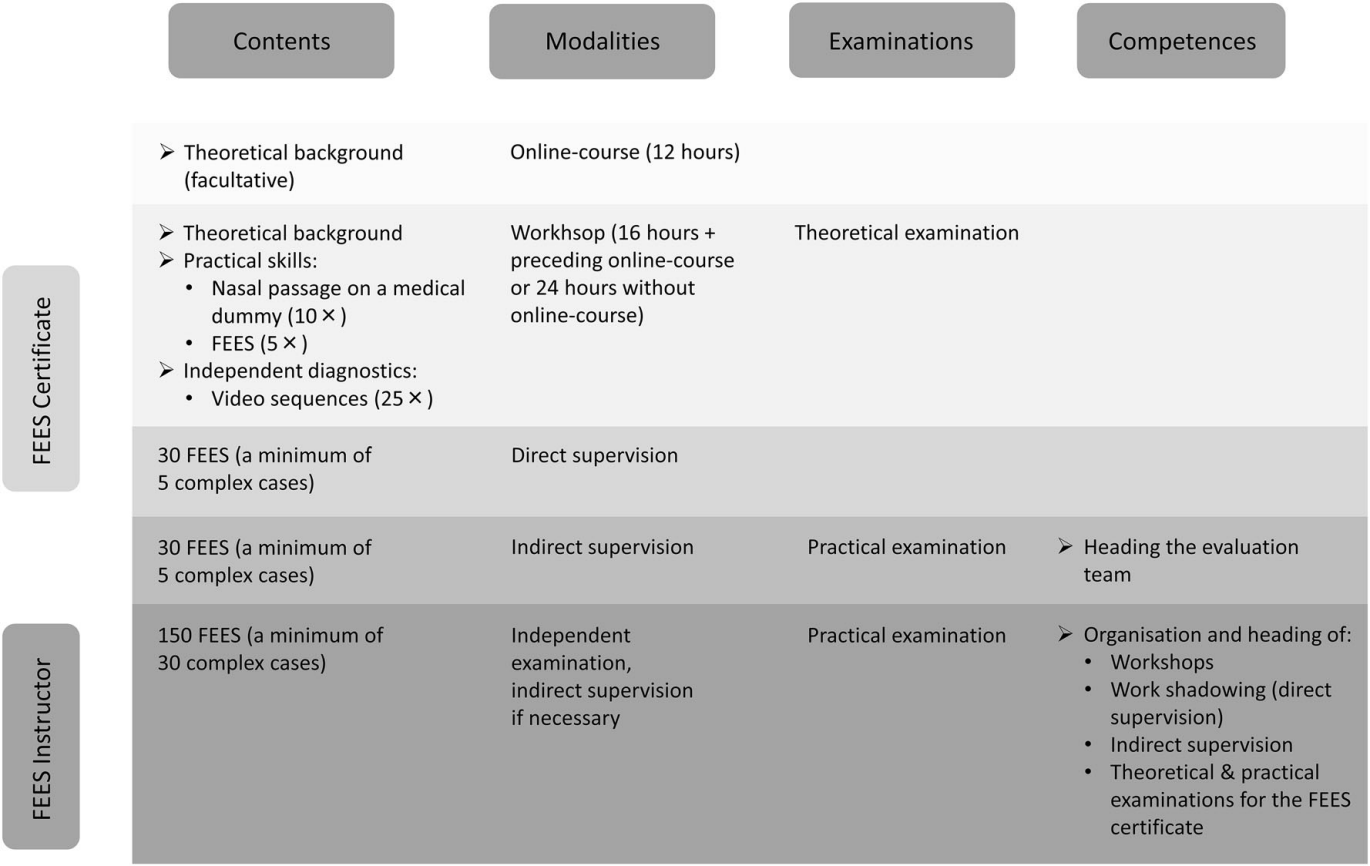
Detailed overview of educational steps leading to the FEES certificate and FEES instructor certificate

Training consists of the following sections (Fig. 1):

- Either
  - 12 h online education module and 16 h on-site Workshop
- Or
  - 24 h on-site Workshop
- Plus
  - FEES practice under direct supervision and
  - FEES practice under indirect supervision

#### Online Learning

Twelve hours online learning module on pathophysiology, diagnosis, and management of OD and its complications will be provided. Graduates of ESSD Postgraduate Diploma on Swallowing Disorders, www.postgraduateswallowingdisorders.com, do not need to do this step.

#### Workshop

The workshop consists of 16 hours of training in theoretical and practical knowledge (the on-site workshop will be 24 h if the 12 h online module is not implemented). The theoretical topics covered are listed in Table 1. Handling of the endoscope will be practiced (a minimum of ten times) using a medical dummy. The participants will then improve their technical skills by means of reciprocal examination (a minimum of five times). Interpretation of typical endoscopic findings will be practiced using suitable video sequences. Each participant will analyze at least 25 sequences independently. At the end of the workshop candidates will have to pass a theoretical exam on the online and on-site content.

**Table 1:** Contents of the basic workshop

(A) Basics
History of FEES
Aims of the evaluation
Indications
Contraindications
Limits
Examination procedure
Distribution of tasks and responsibilities within the examination team
Alternative instrumental dysphagia assessments and their indications
Videofluoroscopic swallow study
Pharyngeal and esophageal manometry
(B) Diseases
Neurovascular diseases (e.g. ischemic stroke)
Neurodegenerative diseases (e.g. Parkinson’s disease)
Neuromuscular diseases (e.g. ALS, polymyositis)
Neurotraumatology (e.g. traumatic brain injury)
Neuro-oncological diseases (e.g. gliomas, paraneoplastic diseases)
Neuroinfectious diseases (e.g. brainstem encephalitis)
Syndrome disorders (e.g. Down syndrome, Rett syndrome)
Age-related changes in the swallowing mechanism (presbyphagia, sarcopenia, malnutrition)
Mental impairment (e.g. congenital brain defects)
Multi-morbidity (polypharmacy, frailty, adverse drug reactions)
Differential diagnosis of neurogenic dysphagia (e.g. cervical spine surgery, Morbus Forestier, disobliteration of the internal carotid artery, laryngeal reflux, Zenker’s diverticulum)
(C) Equipment
Flexible endoscope
Fiber endoscope
Video endoscope
Light source
Video camera
Processing software
Consumables
Hygiene and cleansing
(D) Preparations
Patient information
Patient positioning
Local anesthesia
Nasal decongestant
Defogging
Emergency management
(E) Endoscope handling and placement
Holding and operating the endoscope
Nasal passage
Velum
Oropharynx/hypopharynx and larynx
Home position
Close view
(F) Standard FEES protocol
Anatomic observation
Stenosis of the nasal meatus
Velopharyngeal incompetence
Pharyngeal stenosis
Post-operative and post-chemo/radiotherapy findings
Mucosal abnormalities
Hypertrophic base of the tongue
Edema
Signs of gastro-esophageal reflux
Irregular position of gastric tube
Saliva pooling
Abnormal position of epiglottis, arytenoid cartilage, and glottis
Physiological examination
Velopharyngeal closure
Movement of the base of the tongue
Epiglottis inversion
Pharyngeal wall contraction
Vocal cord and vestibular fold movement
Sensory functions
Airway
Evaluation of swallowing
Choice of consistency depending on the problem at hand
‘White-out’ characterization and post-swallow stage
Identification of the salient findings and use of validated scales
Oral bolus control, leaking
Delayed swallowing reflex
Residues
Penetration
Aspiration
Temporal characteristics of penetration and aspiration (predeglutitive, intradeglutitive or postdeglutitive)
Adequacy of clearance effort and sensory feedback
Regurgitation
Identification of the main pathomechanisms
Evaluation of different therapeutic maneuvers
Evaluation and interpretation of the examination
Classification
Degrees of severity
Therapeutic consequences (e.g. nutrition management, rehabilitation)
Communication of results of dysphagia assessment, education of patients and relatives
Indications for referral to further medical departments (e.g. otolaryngology, gastroenterology, phoniatrics)
(G) Disease-specific examination protocols
FEES protocol for stroke patients
FEES tensilon test
Fatigable swallowing test
FEES l-dopa test
Decannulation protocol

#### FEES Under Direct Supervision

The second phase involves performing FEES under direct supervision in a hospital. Handling of the endoscope as well as planning of the interventions will be practiced during 30 explorations, and concise reports of the findings will be prepared in each individual case. These will include standard cases as well as a minimum of five complex cases. The latter will include patients with compromised respiratory function, tracheotomized patients, patients whose ability to cooperate is impaired due to ailments such as aphasia or an acute confusional state, as well as patients displaying motor restlessness caused by, for example, a movement disorder (Table 2). It is the responsibility of the participants to find and contract a clinic where they can do this practice. ESSD will help them find a supervisor in the country they work in but participants need to have permission to practice in hospitals.

**Table 2:** Characteristics of complex patients

Respiratory impairment
Tracheostomy
Restlessness (Parkinson’s disease, dystonia, delirium)
Limited understanding of the situation (severe aphasia due to stroke or encephalitis)
Fluctuating state of consciousness

#### FEES Under Indirect Supervision

During the last stage of the program, 30 FEES will be performed independently and documented in the training record book. At least 5 will involve complex cases. The supervisor from the previous stage will be available for questions and will also discuss critical findings with the trainee.

**Fig. 2:**
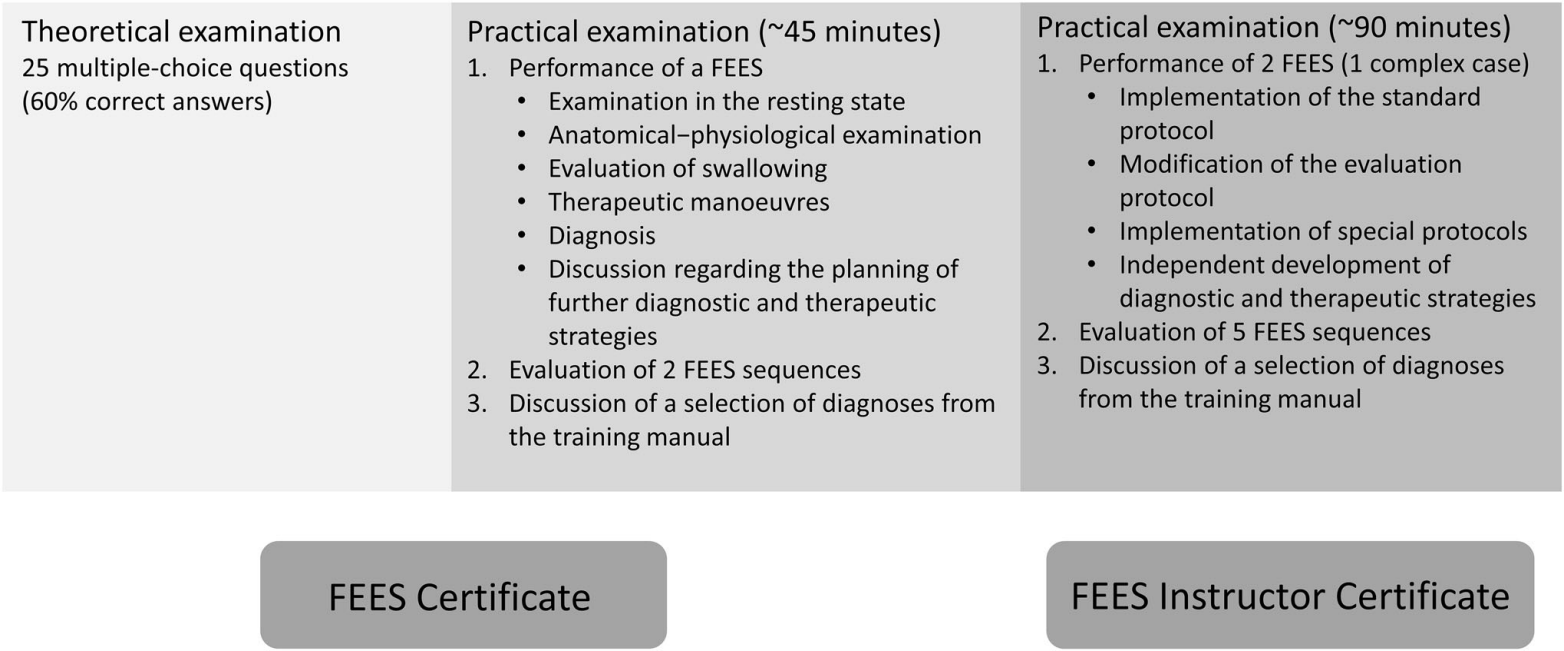
Examination components of the FEES certificate and FEES instructor certificate

The education ends with a practical examination which involves performing FEES independently. In addition, the examinee should write a systematic report, consider additional diagnostic steps where necessary, and make suggestions regarding further management. Ideally, detailed rehabilitation planning should be provided in the context of a multidisciplinary team. The test also comprises assessment and diagnosis of three additional FEES sequences prepared by the examiner. Finally, selected findings recorded during the training period are discussed with the examinee (Fig. 2).

### FEES Instructor Certificate

The FEES instructor possesses more profound knowledge and skills. He/she has the ability to independently assess all cases, including complex ones. He/she is licensed to organize FEES-training seminars, can offer work-shadowing opportunities and is entitled to administer the theoretical and practical FEES-certificate examinations in agreement with the ESSD. Key learning objectives of this educational step include (i) to safely pass the scope in difficult clinical situations, (ii) to adapt the FEES procedure to the given clinical situation and to use disease-specific protocols, (iii) to explain the pathophysiology of observed important findings, (iv) to identify subtler pathological findings, (v) to make more accurate suggestions regarding further management in the multidisciplinary team context.

To become a FEES instructor, additional systematic practical training is required that comprises a minimum of 150 FEES, 30 of which must pertain to complex cases. These evaluations, including complications, must be documented in the FEES training record book. Difficult diagnoses are to be discussed with the instructor.

At the end of this educational period, a practical examination will be taken in a hospital. This test includes two FEES, one of which must be a complex case. Besides implementing the standard FEES protocol, the examinee must also adapt the procedure as needed without external help, explain his examination steps and be able to implement special FEES protocols (such as the FEES-Tensilon-Test or the FEES-Levodopa-Test). The examinee must also be able to develop diagnostic and therapeutic strategies without assistance. Additionally, he must assess five video sequences prepared by the examiner. Findings documented throughout the preceding training period will be discussed during the examination (Table 2). The candidate must also be able to explain and substantiate the FEES routine established in his institution using appropriate documents (such as diagnosis forms, clinical algorithms).

**Fig. 3:**
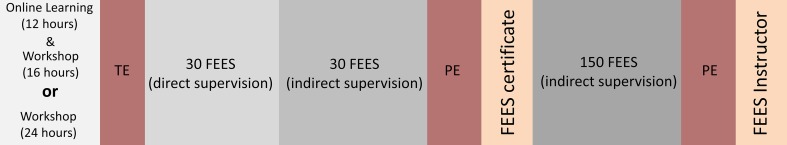
Components of the FEES certificate and FEES instructor certificate. *TE* theoretical examination, *PE* practical examination

The complete FEES-training curriculum is summarized in Fig. 3.

Regardless of the level of training, the required endoscopies can be performed in the candidate’s own institution and/or within the scope of work-shadowing opportunities and workshops in external institutions. Work shadowing is especially meaningful during the initial stage of training, during which the mediation of technical skills, requiring intensive personal supervision, is particularly important. For advanced users, workshops offering discussions on complex cases could be an option, as these are an ideal setting in which to discuss rare, subtle or difficult-to-interpret findings in a focused manner.

## Training Record Book

Complete documentation of the FEES education in the training record book is required.

## Task Assignment and Delegation

As stated above, this accreditation program is also open to allied health care professionals, a group of non-medical professionals. For this reason, the aspects of task assignment and delegation are briefly addressed here. In principle, this curriculum encourages the performance of FEES by a multidisciplinary team including physicians and allied health care professionals. It is the conviction of the ESSD that, within the FEES team, tasks should be assigned flexibly, taking into account the training level of each person involved. However, it is important to note that the FEES certificate described here does not overrule national regulations. Therefore, holders of either the FEES certificate or the FEES instructor certificate do not automatically acquire the right to carry out FEES in their home countries as this depends on national regulations. What the ESSD accredits is that the skills and the knowledge to perform FEES in neurologic and geriatric patients have been achieved, paving the way for a claim for professional recognition to widen scope of practice. Whether, and under which conditions, non-medical professionals are allowed to do FEES is defined by country-specific legal regulations. Therefore, it is suggested that professionals with an interest in this education should carefully check those regulations of their home countries before registering.

## Applying for the FEES Certificate and the FEES-Instructor Status

Following completion of the different educational steps of this curriculum, requests for the FEES certificate and the FEES-instructor status can be submitted to the ESSD FEES accreditation board either by e-mail or regular mail (for further information with regards to the accreditation board see below).

## Transitional Arrangement

Until 31 December 2018, the FEES certificate and the FEES instructor status, including full entitlement to administer examinations leading to the FEES instructor status, can be granted within the framework of a transitional arrangement under the conditions listed below.

Qualifications required for the FEES certificate:

- Proof of training in an institution with FEES expertise
- 2 years of experience in the area of FEES with patients presenting OD
- A minimum of 200 performed evaluations
- Passing a written onsite exam provided by the accreditation board.

Qualifications required for the FEES instructor certificate:

- Five years of experience in the area of FEES with patients presenting OD
- A minimum of 500 performed evaluations
- Establishment of examination standards within the applicant’s hospital
- Internal advanced training for staff members
- For physicians specialist title
- Passing a written onsite exam provided by the FEES accreditation board.

Exceptionally, holders of comparable certificates from other societies or professions that include the training of FEES and management of swallowing disorders, such as otorhinolaryngologists and phoniatricians, may be granted the FEES certificate or the FEES instructor certificate upon individual application and without sitting an exam.

All decisions with regards to the transitional arrangement will be taken by the accreditation board of the ESSD.

## Accreditation of Curricular FEES Training Courses

FEES certificate training events planned by FEES instructors must be evaluated and accredited by the accreditation board. Instructors offering courses with ESSD accreditation should be members of the ESSD.

## Organization and Implementation

All organizational aspects will be managed by the **ESSD FEES accreditation board**. All involved medical disciplines as well as health care professionals will be represented on this board. The following tasks are confined to the accreditation board:

- Reviewing applications for the FEES certificate and the FEES instructor certificate within the transitional arrangement
- Reviewing regular applications for the FEES certificate and the FEES instructor certificate
- Reviewing and accrediting curricular FEES training courses
- Evaluating web-based online material used for FEES courses
- Assigning FEES trainees to FEES instructors for practical examinations
- Looking for supervisors in the country of the trainees
